# Trajectory of blood pressure after initiating anti-calcitonin gene-related peptide treatment of migraine: a target trial emulation from the veterans health administration

**DOI:** 10.1186/s10194-023-01640-y

**Published:** 2023-08-15

**Authors:** Kaicheng Wang, Brenda T. Fenton, Vinh X. Dao, Alexander B. Guirguis, Sarah E. Anthony, Melissa Skanderson, Jason J. Sico

**Affiliations:** 1 Department of Veterans Affairs, Research, Education, Evaluation and Engagement Activities Center for Headache, Headache Centers of Excellence, Orange, CT USA; 2 Yale Center for Analytical Sciences, Yale School of Public Health, 300 George St STE 511, New Haven, CT 06511 USA; 3 Pain Research, Informatics, Multi-Morbidities, and Education Center, VA Connecticut Healthcare System, West Haven, CT USA; 4 Pharmacy Benefits Management Services, VA Minneapolis Health Care System, Minneapolis, MN USA; 5 Headache Center of Excellence, VA Minneapolis Health Care System, Minneapolis, MN USA; 6 Pharmacy Benefits Management Services, VA Connecticut Healthcare System, West Haven, CT USA; 7 Headache Center of Excellence, VA Connecticut Healthcare System, West Haven, CT USA; 8 Department of Neurology, Yale School of Medicine, New Haven, CT USA; 9 Department of Internal Medicine, Yale School of Medicine, New Haven, CT USA

**Keywords:** Anti-CGRP, Blood pressure, Hypertension, Migraine

## Abstract

**Background:**

Calcitonin gene-related peptide (CGRP) is involved in migraine pathophysiology and blood pressure regulation. Although clinical trials have established the cardio-cerebrovascular safety profile of anti-CGRP treatment, limited high-quality real-world evidence exists on its long-term effects on blood pressure (BP). To address this gap, we examined the safety of anti-CGRP treatment on BP in patients with migraine headache in the Veterans Health Administration (VHA).

**Methods:**

We emulated a target trial of patients who initiated anti-CGRP treatment or topiramate for migraine prevention between May 17th, 2018 and February 28th, 2023. We calculated stabilized inverse probability weights to balance between groups and then used weighted linear mixed-effect models to estimate the systolic and diastolic BP changes over the study period. For patients without hypertension at baseline, we estimated the cumulative incidence of hypertension using Kaplan–Meier curve. We also used weight mixed-effect Poisson model to estimate the number of antihypertension medications for patients with hypertension at baseline.

**Results:**

This analysis included 69,589 patients and 554,437 blood pressure readings. of these, 18,880 patients received anti-CGRP treatment, and they were more likely to be women, have a chronic migraine diagnosis and higher healthcare utilization than those received topiramate. Among patients without hypertension at baseline, we found no significant differences in systolic BP changes over the four-year follow-up between anti-CGRP (slope [standard error, SE] = 0.48[0.06]) and topiramate treated patients (slope[SE] = 0.39[0.04]). The incidence of hypertension was similar for anti-CGRP and topiramate group (4.4 vs 4.3 per 100 person-years). Among patients with hypertension at baseline who initiated anti-CGRP treatment, we found a small but persistent effect on exacerbating hypertension during the first four years of treatment, as evidenced by a significant annual 3.7% increase in the number of antihypertensive medications prescribed (RR = 1.037, 95%CI 1.025–1.048).

**Conclusions:**

Our findings suggest that anti-CGRP treatment is safe regarding blood pressure in patients without hypertension. However, for those with baseline hypertension, anti-CGRP treatment resulted in a small but persistent increase in the number of antihypertensives, indicating an exacerbation of hypertension. Future studies are needed to evaluate the cardio-cerebrovascular safety of anti-CGRP treatment beyond the first four years.

**Supplementary Information:**

The online version contains supplementary material available at 10.1186/s10194-023-01640-y.

## Background

Calcitonin gene-related peptide (CGRP) is a neuropeptide that plays a role in both migraine pathophysiology and blood pressure regulation. Blocking endogenous CGRP could be risky for patients with hypertension [1, 2]. However, randomized clinical trials and open-label extension studies have not found any significant adverse effects on blood pressure or an increased risk of cardio-cerebrovascular diseases from using CGRP monoclonal antibodies (mAb) or small molecular antagonists [3–5]. The incidence rate of erenumab-related hypertension was 0.144 per 100 person-years based on postmarking surveillance from May 2018 to January 2020 [6]. During the same period, the U.S. Food and Drug Administration Adverse Event Reporting System identified 61 cases of elevated blood pressure, leading to a warning of hypertension being amended to the prescribing information for erenumab [7]. The lack of standard care controls, loss of follow-up, and reporting bias in FAERS and open-label extension studies as well as the exclusion of patients who were at risk of acute or serious cardio-cerebrovascular disease [8, 9], and, in some cases, with uncontrolled hypertension from clinical trials [10, 11], raised concerns about the validity of determining the overall safety of anti-CGRP treatment. High-quality real-world evidence on the effect of anti-CGRP treatment on blood pressure is scarce. To date, the only observation study that reported a 5.2 mm Hg increase in systolic blood pressure (BP) among 196 patients treated with erenumab and fremanezumab did not include a direct comparison group within the same model or adjust for potential confounders [12].

Given these equivocal findings, we analyzed electronic health record data from the Veterans Health Administration (VHA) between May 17^th^, 2018 and February 28^th^, 2023 to investigate the trajectory of blood pressure in veterans with migraine after initiating erenumab, fremanezumab, galcanezumab, rimegepant or atogepant for migraine prevention in this national integrated health system.

## Methods

### Specification and emulation of the target trial

We emulated this target trial to determine the effect of anti-CGRP treatment on systolic and diastolic BP among patients with migraine disorder who were either free from or diagnosed with hypertension at baseline, and compared these individuals to patients who received topiramate, the most commonly prescribed migraine preventive within the VHA, known to have minimal effect on blood pressure between May 17^th^, 2018 (approval of erenumab), and February 28^th^, 2023. Supplemental Table 1 summarizes the key protocol components for the target trial. We identified patients who had been diagnosed with migraine disorder from the VHA Headache Centers of Excellence administrative data cohort between October 1^st^, 2008, to September 30^th^, 2022 using International Classification of Disease diagnostic codes [13]. This administrative data cohort included over half a million patients with migraine disorder who were diagnosed and treated within the VHA. To minimize measurement errors, we included patients who had regular contacts with the VHA, defined as having at least one outpatient encounter with BP measured prior to the migraine preventive treatment. The treatment strategies in the target trial were initiated CGRP mAbs and small molecule CGRP antagonists (Supplemental Table 2), or initiation of topiramate for migraine prevention. Eptinezumab was not dispensed through outpatient pharmacy, therefore was not included in this study. Rimegepant for migraine prevention was defined as receiving 12 or more tablets per 30 days. Of note, patients who were prescribed topiramate for migraine prevention and rimegepant or ubrogepant for acute treatment were excluded from the study.

We classified eligible individuals into two arms as if they were randomly assigned to a treatment strategy conditional on the baseline covariates: age, gender, race, ethnicity, VA service connection disability status, rurality, smoking status, body mass index (BMI); migraine characteristics such as years since coded migraine diagnosis, chronic migraine diagnosis, number of headache-related primary care, emergency room and neurology outpatient encounters within one year prior to the initial prescription; triptan and migraine preventive medications such as other anticonvulsants (lamotrigine, pregabalin and valproates), angiotensin-converting enzyme inhibitor or angiotensin II receptor blocker (ACEI/ARB: lisinopril and candesartan), β-blockers (atenolol, bisoprolol, metoprolol, nadolol, propranolol and timolol), tricyclic antidepressants (amitriptyline, nortriptyline) and neurotoxins (abobotulinumtoxinA, incobotulinumtoxinA and onabotulinumtoxinA) [14]; and baseline diagnosis of hypertension.

The primary outcome of interest was the repeated blood pressure measurements taken by a nurse during outpatient encounters. We used the average BP if there were multiple readings for a same encounter. Additionally, patients who did not have blood pressure measured during the follow-up period were excluded from the analysis. For patients who had no hypertension at baseline, BP readings after the subsequent diagnosis of hypertension were omitted. The secondary outcome of interest was time to a hypertension diagnosis. For patients who already had hypertension at baseline, the secondary outcome was the change in number of antihypertensive medications after initiating anti-CGRP treatment or topiramate. Patients were followed from the date of initial prescription (baseline), until the dispense date of last prescription plus the number of days supplied of the prescription regardless of treatment interruption or within-class switch between fills, loss of follow-up (defined as the last encounter date), or administrative end of follow up by February 28^th^, 2023. The medication possession ratio (MPR) was calculated by summing of days’ supply of all prescriptions and divided by the number of days from baseline to the end of treatment and truncated at the maximum value of 1.0.

Other comorbidities related to hypertension were also obtained from VHA Corporate Data Warehouse, which included alcohol-related disorder, chronic kidney disease, diabetes mellitus, hyperlipidemia and obstructive sleep apnea. Outpatient pharmacy data was also reviewed for medications with antihypertensive effect, which was categorized into 11 classes: ACEIs/ARBs, α-blockers, β-blockers, calcium channel blockers, centrally acting sympathetic agonist, loop diuretics, thiazide and thiazide-like diuretics, potassium-sparing diuretics, direct renin blocker, nitrates and vasodilators [15]. Because the administrative data could not differentiate the exact indication for a prescription, some medications, such as lisinopril and metoprolol, could be classified as both migraine preventive and antihypertensive medications. Missing values of covariates were coded as “missing/unknown”, respectively.

### Statistical analysis

Baseline characteristics of study sample were summarized as frequency and percentage, mean and standard deviation (SD) or median and interquartile range (IQR) as appropriate. The intention-to-treat analysis examined the association of initiating anti-CGRP treatment versus topiramate on blood pressure. Estimating the observational analog requires adjustment for baseline confounders. First, we estimated the stabilized inverse probability weight (IPW) for each individual in the study sample. The denominator of the stabilized weight was the probability (propensity score) that individuals received anti-CGRP treatment given their baseline confounders, and it was estimated from a multivariate logistic regression model. The numerator of the stabilized weight was the observed probability of receiving anti-CGRP treatment. We used standardized mean difference to assess the balance between groups, with a value less than 0.1 indicating good balance (Supplemental Fig. 1).

Next, weighted linear mixed-effect models were built using all available blood pressure measures after baseline to estimate the change overtime and the difference between groups, while adjusting for fixed effect of baseline SBP or DBP, a natural spline function of follow-up time with 2 degrees of freedom, an interaction between time and treatment group, baseline age, gender, race, BMI, smoking status, obstructive sleep apnea, chronic kidney disease, hyperlipidemia and diabetes, and a random intercept of the patient. For patients who did not have hypertension at baseline, A Kaplan–Meier curve was used to estimate the cumulative incidence of hypertension and the difference between treatment groups was compared using log-rank test. For patients who had hypertension at baseline, weighted mixed-effect Poisson regression was used to model the log of number of antihypertension medications adjusting with a natural spline function of follow-up time with 2 degrees of freedom, an interaction between time and treatment, and baseline characteristics. All statistical analyses were performed using R version 4.2.2 (R Foundation for Statistical Computing, Austria). Statistical significance was considered as a two-tailed *p*-value less than 0.05.

## Results

### Description of the overall sample and comparison of two treatment groups

**Fig. 1:**
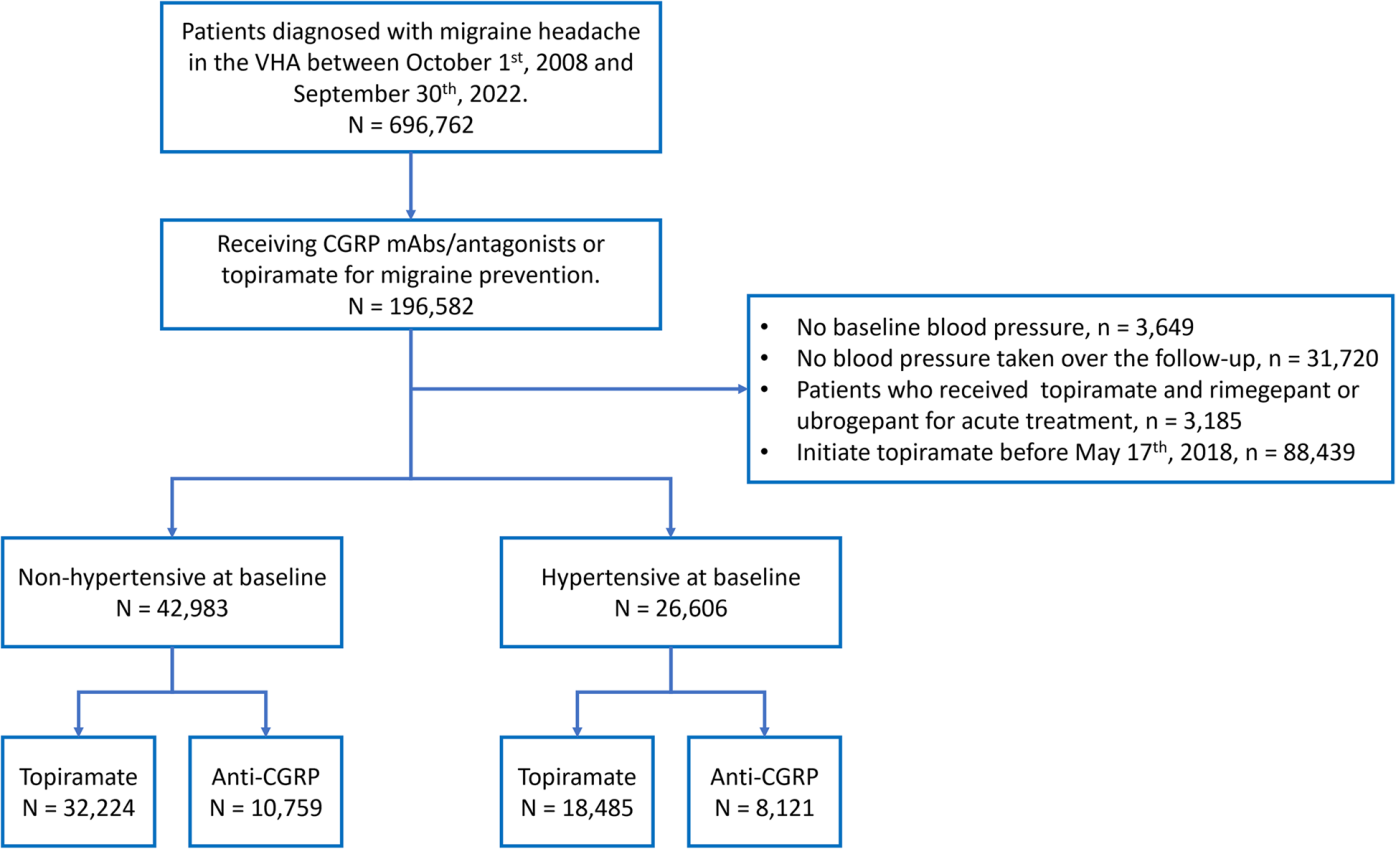
Flowchart. Abbreviations: VHA, Veterans Health Administration; CGRP, calcitonin gene-related peptide; mAbs, monoclonal antibodies

The study flowchart is depicted in Fig. 1. The initial population comprised of 696,762 patients with migraine headache. Out of these patients, 38.9% were ever prescribed anticonvulsants, 19.7% were prescribed ACEI/ARB, 33.3% received β-blockers, 20.8% received TCAs and 3.3% received anti-CGRP treatment for migraine prevention. After exclusion, 69,589 patients were included in this analysis, who had a total of 554,437 blood pressure readings taken between May 17^th^ 2018 and February 28^th^ 2023. The median follow-up length for the entire study sample was 0.9 (IQR 0.3–2.0) year.

In total, 18,880 (27.1%) patients received anti-CGRP treatment (Table 1). Patients received an anti-CGRP treatment had a higher proportion of women (43.1% vs. 34.1%) and persons of white race (65.8% vs. 56.3%) than the comparison group who received topiramate. They were more frequently diagnosed with chronic migraine (53.2% vs. 16.6%) and were prescribed triptans (76.8% vs. 43.8%). Additionally, they had higher healthcare utilization with an average of 2.3 headache-related outpatient neurology visits in the past year. Before starting anti-CGRP treatment, these patients had tried an average of 2.4 classes of conventional migraine preventives. The median MPR was 0.85 (Interquartile range [IQR] 0.55–1.00) for the topiramate group and 0.91 (IQR 0.66–1.00) for the anti-CGRP treatment group.

**Table 1:** Baseline characteristics of 69,589 patients who initiated topiramate or anti-CGRP treatment for migraine prevention between May 17th 2018 and February 28th 2023

	**Non-hypertensive**		**Hypertensive**	
	**Anti-CGRP**	**Topiramate**	**Anti-CGRP**	**Topiramate**
	**(*n* = 10,759)**	**(*n* = 32,224)**	**(*n* = 8,121)**	**(*n* = 18,485)**
Age, mean (SD), y	43.4 (10.5)	40.7 (10.6)	54.4 (11.2)	53.6 (11.8)
Gender ^a^, women (%)	5,507 (51.2)	12,907 (40.1)	2,623 (32.3)	4,394 (23.8)
Race ^a^ (%)				
White	7,214 (67.1)	18,403 (57.1)	5,209 (64.1)	10,122 (54.8)
Black	2,218 (20.6)	9,327 (28.9)	2,206 (27.2)	6,524 (35.3)
Asian	147 (1.4)	690 (2.1)	90 (1.1)	278 (1.5)
Others/Unknown ^b^	1,180 (11.0)	3,804 (11.8)	616 (7.6)	1,561 (8.4)
Ethnicity ^a^, non-Hispanics (%)	9,576 (89.0)	28,026 (87.0)	7,485 (92.2)	17,013 (92.0)
Service connection, yes (%)	10,370 (96.4)	30,641 (95.1)	7,530 (92.7)	16,600 (89.8)
Rurality (%)				
Urban	7,616 (70.8)	23,796 (73.8)	5,579 (68.7)	12,676 (68.6)
Rural	3,122 (29.0)	8,320 (25.8)	2,537 (31.2)	5,769 (31.2)
Unknown	21 (0.2)	108 (0.3)	5 (0.1)	40 (0.2)
Smoking status (%)				
Never	5,711 (53.1)	16,125 (50.0)	3,976 (49.0)	8,045 (43.5)
Current	3,083 (28.7)	10,097 (31.3)	2,370 (29.2)	6,528 (35.3)
Former	1,921 (17.9)	5,630 (17.5)	1,770 (21.8)	3,885 (21.0)
Unknown	44 (0.4)	372 (1.2)	5 (0.1)	27 (0.1)
BMI, mean (SD), kg/m^2^	30.2 (5.8)	30.7 (5.8)	32.5 (6.2)	33.0 (6.4)
Underweight/Normal	1,912 (17.8)	4,797 (14.9)	752 (9.3)	1,511 (8.2)
Overweight	3,650 (33.9)	10,712 (33.2)	2,208 (27.2)	4,897 (26.5)
Obese	5,054 (47.0)	16,096 (50.0)	5,145 (63.4)	12,013 (65.0)
Unknown	143 (1.3)	619 (1.9)	16 (0.2)	64 (0.3)
Systolic BP, mean (SD), mm Hg	121.8 (12.8)	122.6 (12.8)	130.1 (15.4)	131.7 (15.6)
Diastolic BP, mean (SD), mm Hg	77.5 (8.9)	77.3 (9.0)	80.3 (9.7)	80.8 (10.1)
Years since onset of migraine, mean (SD)	5.0 (4.2)	1.9 (3.1)	6.4 (4.5)	3.0 (4.0)
Chronic migraine (%)	5,507 (51.2)	5,170 (16.0)	4,545 (56.0)	3,250 (17.6)
Headache-related encounters in the past year				
Primary care, mean (SD)	1.2 (1.3)	0.9 (1.0)	1.2 (1.5)	0.8 (1.0)
Emergency room, mean (SD)	0.2 (0.9)	0.1 (0.4)	0.2 (1.1)	0.1 (0.5)
Neurology, mean (SD)	2.3 (2.3)	0.4 (0.9)	2.4 (2.4)	0.5 (1.0)
Prescribed Triptans (%)	8,638 (80.3)	14,624 (45.4)	5,860 (72.2)	7,591 (41.1)
History of migraine preventives, ever				
No. of medications, mean (SD)	2.1 (1.4)	0.5 (0.8)	2.8 (1.6)	1.1 (1.2)
Other Anticonvulsants ^c^ (%)	4,870 (45.3)	3,292 (10.2)	4,392 (54.1)	3,042 (16.5)
ACEI/ARB (%) ^d^	262 (2.4)	324 (1.0)	3,099 (38.2)	4,540 (24.6)
β-blockers (%) ^d^	5,034 (46.8)	4,522 (14.0)	5,201 (64.0)	5,844 (31.6)
TCAs (%)	4,625 (43.0)	4,355 (13.5)	3,742 (46.1)	3,023 (16.4)
Neurotoxins (%)	4,485 (41.7)	761 (2.4)	3,554 (43.8)	495 (2.7)
Alcohol-related disorder (%)	1,790 (16.6)	5,575 (17.3)	1,868 (23.0)	4,706 (25.5)
Chronic kidney disease (%)	188 (1.7)	277 (0.9)	798 (9.8)	1,506 (8.1)
Diabetes (%)	594 (5.5)	1,386 (4.3)	2,677 (33.0)	5,854 (31.7)
Hyperlipidemia (%)	3,856 (35.8)	8,606 (26.7)	5,926 (73.0)	12,608 (68.2)
Obstructive sleep apnea (%)	3,532 (32.8)	7,491 (23.2)	4,751 (58.5)	8,788 (47.5)
Medications with antihypertensive effect				
No. of medications, mean (SD)			1.5 (1.3)	1.4 (1.3)
ACEIs/ARBs (%) ^d^			3,062 (37.7)	7,334 (39.7)
α-blockers (%)			1,232 (15.2)	2,464 (13.3)
β-blockers (%) ^d^			3,094 (38.1)	5,223 (28.3)
Calcium channel blockers (%)			2,198 (27.1)	4,666 (25.2)
Centrally acting sympathetic agonist (%)			181 (2.2)	324 (1.8)
Diuretics, potassium-sparing (%)			398 (4.9)	752 (4.1)
Diuretics, thiazide (%)			1,634 (20.1)	4,135 (22.4)
Diuretics, loop (%)			382 (4.7)	776 (4.2)
Nitrates (%)			171 (2.1)	401 (2.2)
Direct renin blocker (%)			0 (0.0)	2 (< 0.1)
Vasodilators (%)			134 (1.7)	262 (1.4)
Follow-up, Median (IQR), y	1.1 (0.4–2.2)	0.8 (0.2–1.8)	1.0 (0.4–2.2)	0.9 (0.2–2.1)
Medication possession ratio, Median (IQR)	0.89 (0.64–1.00)	0.83 (0.53–1.00)	0.92 (0.68–1.00)	0.89 (0.59–1.00)
No. of BP measurements, Median (IQR)	5.0 (3.0–11.0)	4.0 (2.0–8.0)	7.0 (4.0–15.0)	6.0 (3.0–12.0)

*Abbreviations*: *SD* Standard deviation, *BP* Blood pressure, *IQR* Interquartile range

^a^ Gender, race and ethnicity were self-reported from the electronic health record

^b^ Race were self-reported by each person in the administrative dataset and others included Alaska Native or American Indian, Native Hawaiian or other Pacific Islander, and multi-race

^c^ Other Anticonvulsants included lamotrigine, pregabalin and valproates only

^d^ Not mutually exclusive

At baseline, 26,606 (38.2%) patients were diagnosed with hypertension, and they were, on average, 12 years older than patients without hypertension at baseline (53.8[SD: 11.6] vs. 41.4[SD: 10.7] years). They had a lower proportion of women (26.4% vs. 42.8%) and a higher proportion of Black veterans (32.8% vs. 26.9%) and veterans who currently or previously smoked (54.7% vs. 48.3%), were obese (64.5% vs. 49.2%), and diagnosed with hyperlipidemia (69.7% vs. 29.0%), diabetes (32.1% vs. 4.6%), and obstructive sleep apnea (50.9% vs. 25.6%). Their mean blood pressure was 131.2/80.6 mm Hg, which classifies them as having hypertension stage 1 (systolic BP 130–139 mm Hg or diastolic BP 80–89 mm Hg) [16]. On average, they were taking one antihypertensive medication. Patients who had hypertension at baseline (OR = 0.94, 95%CI 0.88–0.99) or took ACEI/ARB for migraine prevention (OR = 0.77, 95%CI 0.70–0.85) were less likely to receive anti-CGRP treatment (Supplemental Table 3).

### Blood pressure trajectory among non-hypertensive patients

**Fig. 2:**
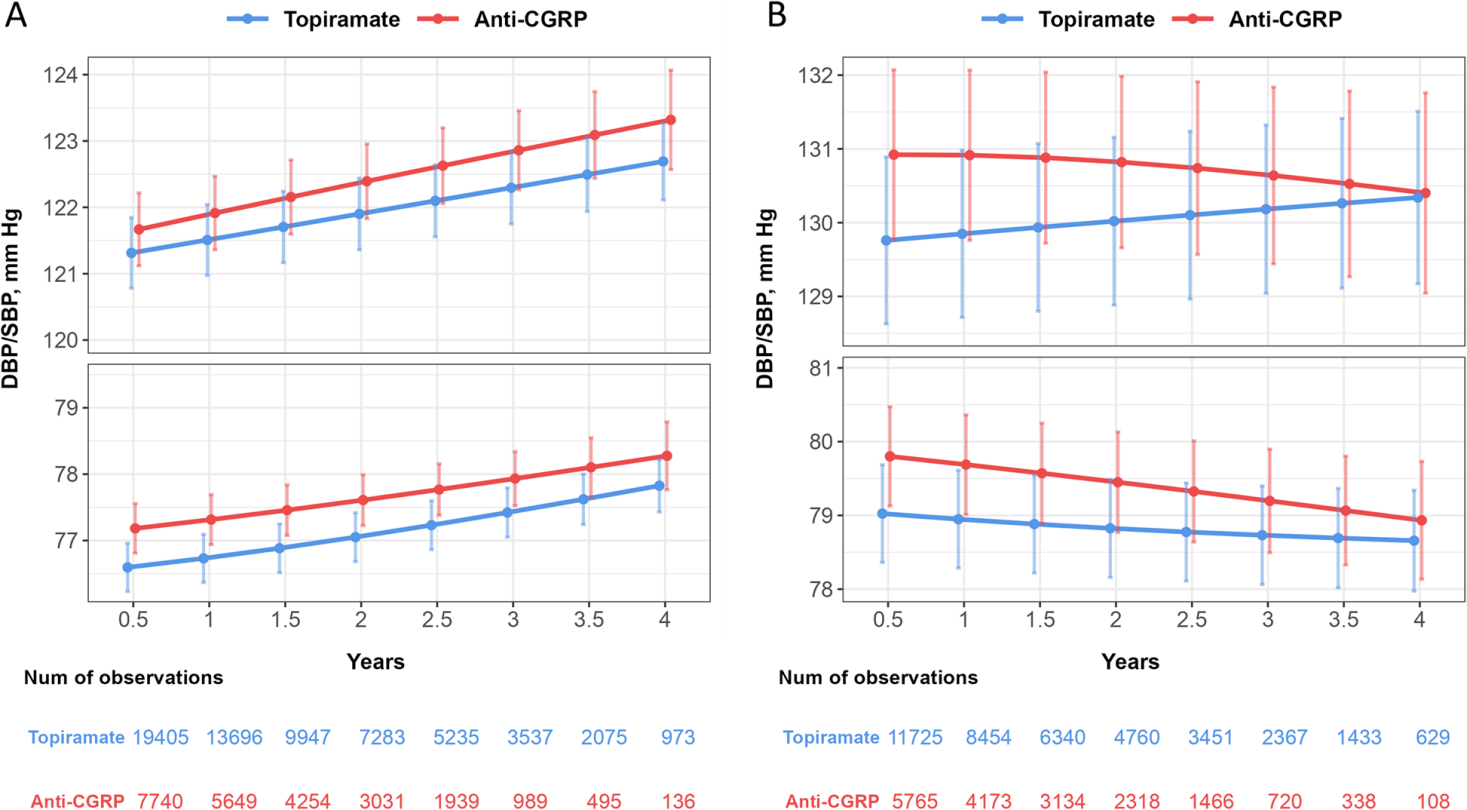
Estimated systolic and diastolic blood pressure trajectories from mixed-effect models among patients who were (**A**) non-hypertensive or (**B**) hypertensive at baseline. Weighted model including fixed effect of treatment group, the natural spline function of time in years, an interaction between treatment group and time, baseline blood pressure, age, gender, race, BMI, smoking status, history of obstructive sleep apnea, chronic kidney disease, hyperlipidemia, diabetes, and random intercept of patient. Abbreviations: CGRP, calcitonin gene-related peptide

**Fig. 3:**
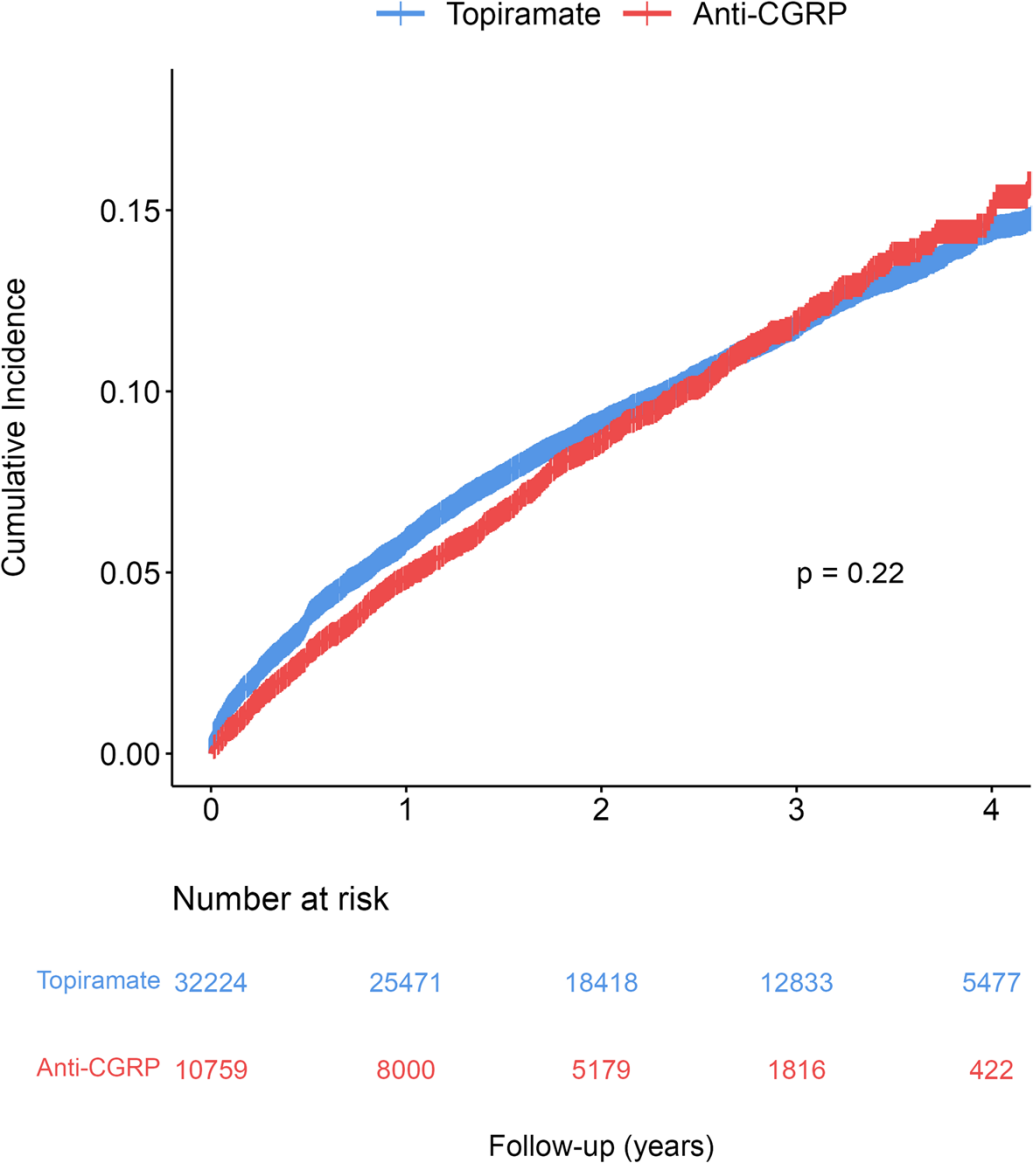
Kaplan–Meier curve on the cumulative incidence of hypertension

The blood pressure readings were estimated using the IPW weighted mixed-effect models, and the resulting trajectories are presented in Fig. 2a. Patients who received anti-CGRP treatment had an estimated SBP of 121.9 mm Hg (95% confidence interval, CI 121.4–122.5) at one year of follow-up, which then increased over the four years period (slope[standard error, SE] = 0.48[0.06], *P* < 0.001). However, this increase was not statistically significant compared to the trajectory of patients who received topiramate (slope[SE] = 0.39[0.04]; Δslope = 0.09, *P* = 0.21). The DBP had a similar trajectory and was 77.3 mm Hg (95%CI 76.9–77.7) for anti-CGRP group at one year after initiation of anti-CGRP treatment. The difference in slope between the anti-CGRP treatment and topiramate was also not statistically significant (slope[SE] = 0.28[0.04] vs. 0.30[0.03], *P* = 0.73). The cumulative incidence of hypertension was plotted using a Kaplan–Meier curve, as shown in Fig. 3. The incidence rate of hypertension following anti-CGRP treatment was 4.4 per 100 person-years, which was not significantly different from the rate for patients treated with topiramate (4.3 per 100 person-years; log-rank test, *P* = 0.22).

### Blood pressure and antihypertensive medication changes among hypertensive patients

**Fig. 4:**
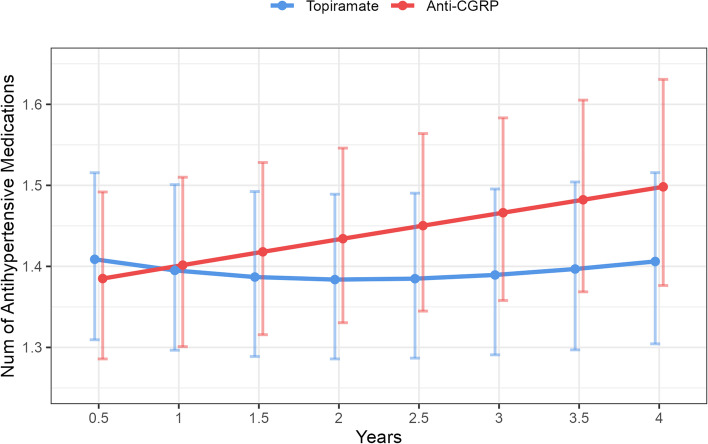
Estimated number of antihypertensive medications from mixed-effect Poisson regression model. Weighted model including fixed effect of treatment group, the natural spline function of time in years, an interaction between treatment group and time, baseline number of antihypertensive medications, age, gender, race, BMI, smoking status, history of obstructive sleep apnea, chronic kidney disease, hyperlipidemia, diabetes, and random intercept of patient

Among patients who had baseline hypertension and initiated anti-CGRP treatment (Fig. 2b), during the first four years of follow-up, systolic blood pressure remained stable (slope [SE] = -0.06[0.08], *P* = 0.46) though had a significantly slower trend compared to those receiving topiramate (Δslope = -0.24, *P* = 0.014); this difference is likely due to an increase in antihypertensive medication use (Fig. 4), as patients on anti-CGRP treatment had an annual 3.7% increase in the number of antihypertensive medications (RR = 1.037, 95%CI 1.025–1.048).

## Discussion

In this retrospective examination of 69,589 patients who were prescribed either anti-CGRP treatment and topiramate for migraine prevention between May 17^th^ 2018 and February 28^th^ 2023 in the VHA, the incidence rate of hypertension was 4.4 per 100 person-years in this cohort, which was twice higher than those reported from open-label extension studies [5, 17] (1.9 and 2.2 per 100 person-years) and postmarketing surveillance [6] (0.144 per 100 person-years). The higher incidence of hypertension may be attributable to a greater burden of vascular risk factors in our real-world sample of veterans (e.g. 30.7% smoking, 49.2% obesity, 29.0% hyperlipidemia) and limitations intrinsic to open label extension studies of clinical trials and voluntary reporting of adverse drug reactions [18, 19]. Additionally, patients with migraine headaches often rely on acetaminophen, non-steroid anti-inflammatory drugs and triptans as acute treatment, which can exacerbate hypertension and increase the risk of secondary cardio-cerebrovascular events [20, 21]. This potential risk may lead healthcare providers to be less inclined to recommend anti-CGRP treatment to patients with high-risk cardio-cerebrovascular profiles. Our initial analysis using the IPW model also supports this reluctance, as patients with a history of hypertension (OR = 0.94, 95%CI 0.88–0.99) or those taking lisinopril or candesartan for migraine prevention (OR = 0.77, 95%CI 0.70–0.85) were less likely to have received anti-CGRP treatment.

Among patients without hypertension at baseline, the changes in systolic or diastolic blood pressure after initiating anti-CGRP treatment were similar to those observed in patients treated with topiramate. Among those with baseline hypertension, we found a small but persistent effect in exacerbating hypertension during the first four years after initiating anti-CGRP treatment, as evidenced by a small but significant annual 3.7% increase in the number of antihypertension medications prescribed throughout the follow-up period, which is similar to the increase observed in patients with overweight BMI compared to those with a normal BMI (RR = 1.037, 95%CI 1.002–1.074). This escalation of antihypertensive medication was not observed in the 12-week clinical trial that compared erenumab and placebo [6].

Understanding the effect of anti-CGRP treatment on blood pressure may help inform clinical decisions and improve cardio-cerebrovascular outcomes in patients with migraine. ACEIs/ARBs and β-blockers are recommended for primary prevention of ischemic stroke or cardiovascular disease in individuals with a high risk of developing or prevalent atherosclerotic disease, and are routinely used for migraine prevention [22, 23]. However, in the VHA, ACEI/ARB (19.7%) and β-blockers (33.3%) were less frequently prescribed than anticonvulsants (38.9%) for migraine prevention, suggesting that providers may be focusing more on prevention of migraine than considering future vascular risks. To the counterpoint, the 2014 American Heart Association/American Stroke Association (AHA/ASA) Primary Prevention of Stroke Guidelines notes, “no proven primary prevention strategy exists for patients with migraine.” However, the AHA/ASA guideline does recommend using a cardiovascular risk calculator to identify that at high-risk of cardio-cerebrovascular events within the next 10 years. As such, providers should consider conducting a comprehensive assessment of cardio-cerebrovascular comorbidities and risk factors when migraine preventive treatment is indicated, and closely monitor the blood pressure changes for the first four years if anti-CGRP treatment is prescribed and adjust the antihypertensive medication regimen accordingly.

This study has several limitations. First, 16% of patients (31,720/196,592) did not have their blood pressure measured during the follow-up due to the COVID-19 pandemic and the transition to telehealth [24]. This missingness, specifically related to in-person appointment, is independent of the outcome (blood pressure) given the exposure and covariates. Therefore, conducting a complete cases analysis among patients with regular contacts with the healthcare system should yield unbiased estimations [25]. Second, we did not account for time-varying treatment and confounders, or censor patients if there was a gap of more than 30 days between anti-CGRP or topiramate refills for statistical simplicity. Although, the overall MPRs were similar (> 0.80) between groups, others methods such as marginal structure model should be considered if time-dependent confounders affected by prior treatment are a concern [26]. Third, the median follow-up time is approximately one year, as we intentionally did not impose a minimal exposure or follow-up to avoid immortal time bias [27]. Additionally, the considerable number of observations (1,109 non-hypertensive and 737 hypertensive patients) and the number of patients at risk (*n* = 5,869) at the fourth-year mark should provide sufficient statistical power for accurate estimation. In addition, we did not perform Cox regression as the proportional hazard assumption was violated, and we found in the Kaplan–Meier curve the cumulative incidence of hypertension in the anti-CGRP treatment group was overtaken by the end of the follow-up. The effect of anti-CGRP treatment on blood pressure and risk of cardio-cerebrovascular events should be reassessed once longer follow-up data are available. Lastly, we did not explore the heterogeneity between antibodies targeting CGRP ligand/receptor or gepants. In our study, some patients received multiple anti-CGRP agents for migraine prevention. A wash-out period of approximately 5 half-lives would have been necessary to analyze the effects of individual agents. However, implementing such period would have resulted in a reduction in sample size and follow-up duration, potentially compromising the statistical power and generalizability of our findings. Additionally, a recent study found a minimal difference of 2.2 mm Hg in SBP changes between patients treated with erenumab or fremanezumab [12], raising questions about whether this discrepancy is coincidental or reflective of distinct molecular mechanisms between antibody targeting the ligand and the CLR/RAMP1 (calcitonin-like receptor/ receptor activity-modifying protein) receptor. Further investigation is needed to explore these differences. Meanwhile, studies shown that the CTR (calcitonin receptor)\RAMP1 receptor also involved in migraine pathophysiology and antagonizing the CTR\RAMP1 receptor did not influence blood pressure [28, 29], suggesting a promising and safer migraine preventive treatment for patients with high risks of developing cardio-cerebrovascular diseases.

## Conclusion

In this retrospective cohort study of 69,589 VHA patients between May 17^th^, 2018 and February 28^th^, 2023, we found no association between initiating anti-CGRP treatment and blood pressure elevation or hypertension diagnosis among non-hypertensive patients. However, for those with baseline hypertension, we observed a small but persistent increase in the number of antihypertensives after initiating anti-CGRP treatment, indicating a potential exacerbation of hypertension. Future studies are necessary to evaluate the cardio-cerebrovascular safety of anti-CGRP treatment beyond the first four years.

### Supplementary Information

**Additional file 1:** **Supplemental Table 1.** Specification and Emulation of a Target Trial Evaluating the Effect of anti-CGRP Treatment on Systolic and Diastolic Blood Pressure Among Patients with Migraine Disorder. **Supplemental Table 2.** Details for anti-CGRP mAbs and antagonist for migraine prevention. **Supplemental Table 3.** Odds Ratios and 95% Confidence Intervals from the Denominator Model Estimating the Inverse Probability of Treatment Weights of Receiving anti-CGRP treatment. **Supplemental Figure 1.** Covariate Balance between anti-CGRP and Topiramate group.

## Data Availability

The data used for this study are available with an approved study protocol by the Department of Veterans Affairs. The data are not publicly available due to regulations and ethics agreements. For more information, please visit https://www.virec.research.va.gov. Statistical code is available by contacting the corresponding author.

## Abbreviations

ACEI: Angiotensin-converting enzyme inhibitor
AHA/ASA: American Heart Association/American Stroke Association
ARB: Angiotensin II receptor blocker
BP: Blood pressure
BMI: Body mass index
CGRP: Calcitonin gene-related peptide
CI: Confidence interval
CLR: Calcitonin-like receptor
CTR: Calcitonin receptor
IPW: Inverse probability weight
IQR: Interquartile range
MPR: Medication possession ratio
OR: Odds ratio
RAMP1: Receptor activity-modifying protein
RR: Relative risk
SD: Standard deviation
VHA: Veterans Health Administration
