# Supplementary material for: Trajectory of blood pressure after initiating anti-calcitonin gene-related peptide treatment of migraine: a target trial emulation from the veterans health administration
Source: J Headache Pain. 2023 Aug 15;24(1):108. doi: 10.1186/s10194-023-01640-y (PMC10426172; doi:10.1186/s10194-023-01640-y)
Supplement: Supplementary file 1 — Additional file 1: Supplemental Table 1. Specification and Emulation of a Target Trial Evaluating the Effect of anti-CGRP Treatment on Systolic and Diastolic Blood Pressure Among Patients with Migraine Disorder. Supplemental Table 2. Details for anti-CGRP mAbs and antagonist for migraine prevention. Supplemental Table 3. Odds Ratios and 95% Confidence Intervals from the Denominator Model Estimating the Inverse Probability of Treatment Weights of Receiving anti-CGRP treatment. Supplemental Figure 1. Covariate Balance between anti-CGRP and Topiramate group. [file 10194_2023_1640_MOESM1_ESM.docx]

**Supplemental Table 1. Specification and Emulation of a Target Trial Evaluating the Effect of anti-CGRP Treatment on Systolic and Diastolic Blood Pressure Among Patients with Migraine Disorder**

| **Protocol component** | **Target trial specification** | **Target trial emulation** |
| --- | --- | --- |
| **Eligibility criteria** | - Diagnosed with migraine disorder - No contraindication and no prior anti-CGRP treatment - Regular healthcare system contact - Potential follow-up - Baseline defined as the first eligibility date | In addition to the target trial specification:   - Regular contact defined as having any encounter with BP measured prior to baseline - Potential follow-up defined as any encounters and BP measured during the study period |
| **Treatment strategies** | - Initiation of anti-CGRP treatment at baseline and continued use until a contraindication - Initiation of topiramate at baseline and continued use until a contraindication | Same as for the target trial |
| **Treatment assignment** | Randomly assigned and aware of assigned strategy at baseline | Classified individuals based on their compatible data at baseline, adjusted for baseline confounders to emulate target trial |
| **Outcome** | Primary outcome:   - Systolic and diastolic blood pressure   Secondary outcome:   - Cumulative incidence of hypertension - Number of antihypertensives | Same as for the target trial |
| **Follow-up** | Starts at baseline and ends at the earliest occurrence of:   - Treatment discontinuation - Loss of follow-up - Administrative end of follow-up (Feb 28^th^, 2023) | In addition to the target trial specification:   - Loss of follow-up defined as the last VHA visit |
| **Causal contrasts** | Intention-to-treat effect | Observational analog of intention-to-treat |
| **Statistical analysis** | - Repeated measurement models for blood pressure - Kaplan-Meier estimation for the hypertension incidence - Random-effect Poisson regression model for number of antihypertensives | Adjust for baseline confounders using inverse probability of treatment weights |

**Supplemental Table 2. Details for anti-CGRP mAbs and antagonist for migraine prevention**

| **Generic Name** | **Dosage Form** |
| --- | --- |
| Atogepant | Atogepant 10mg tab |
| Atogepant | Atogepant 30mg tab |
| Atogepant | Atogepant 60mg tab |
| Erenumab-aooe | Erenumab-aooe 140mg/ml autoinjector, 1ml |
| Erenumab-aooe | Erenumab-aooe 70mg/ml autoinjector, 1ml |
| Erenumab-aooe | Erenumab-aooe 70mg/ml autoinjector, pack, 2 |
| Fremanezumab-vfrm | Fremanezumab-vfrm 225mg/1.5ml autoinjector |
| Fremanezumab-vfrm | Fremanezumab-vfrm 225mg/1.5ml inj,syringe,1.5ml |
| Galcanezumab-gnlm | Galcanezumab-gnlm 120mg/ml inj,pen,1ml |
| Galcanezumab-gnlm | Galcanezumab-gnlm 120mg/ml inj,syringe,1ml |
| Rimegepant | Rimegepant 75mg Tab, oral disintegrating |

**Supplemental Table 3. Odds Ratios and 95% Confidence Intervals from the Denominator Model Estimating the Inverse Probability of Treatment Weights of Receiving anti-CGRP treatment**

|  | **OR (95%CI)** | **P value** |
| --- | --- | --- |
| Age | 1.01 (1.01-1.01) | <0.001 |
| Gender, Women vs. Men | 1.51 (1.44-1.59) | <0.001 |
| Race |  |  |
| *Black vs. White* | 0.64 (0.60-0.68) | <0.001 |
| *Asian vs. White* | 0.48 (0.40-0.59) | <0.001 |
| *Others/Unknown vs. White* | 0.90 (0.83-0.97) | 0.007 |
| Ethnicity, non-Hispanics vs. Hispanics | 1.51 (1.40-1.64) | <0.001 |
| Service connection, Yes vs. No | 1.18 (1.07-1.31) | 0.001 |
| Rurality |  |  |
| *Rural vs. Urban* | 1.14 (1.09-1.21) | <0.001 |
| *Unknown vs. Urban* | 0.73 (0.42-1.25) | 0.25 |
| Smoking status |  |  |
| *Current vs. Never* | 0.71 (0.67-0.75) | <0.001 |
| *Former vs. Never* | 0.97 (0.91-1.03) | 0.37 |
| *Unknown vs. Never* | 1.13 (0.79-1.60) | 0.50 |
| Body mass index |  |  |
| *Overweight vs. Normal/Underweight* | 0.91 (0.84-0.98) | 0.017 |
| *Obese vs. Normal/Underweight* | 0.84 (0.78-0.90) | <0.001 |
| *Unknown vs. Normal/Underweight* | 2.13 (1.72-2.64) | <0.001 |
| Years since onset of migraine | 1.06 (1.06-1.07) | <0.001 |
| Chronic migraine, Yes vs. No | 1.93 (1.84-2.03) | <0.001 |
| Headache-related encounters in the past year |  |  |
| *Primary care, log-transformed* | 1.17 (1.15-1.19) | <0.001 |
| *Emergency room, log-transformed* | 0.95 (0.92-0.98) | 0.003 |
| *Neurology, log-transformed* | 1.71 (1.68-1.74) | <0.001 |
| Prescribed triptans, Yes vs. No | 1.61 (1.52-1.70) | <0.001 |
| History of migraine preventives |  |  |
| *Other anticonvulsants, Yes vs. No* | 2.18 (2.01-2.37) | <0.001 |
| *ACEI/ARB, Yes vs. No* | 0.77 (0.70-0.85) | <0.001 |
| *β-blockers, Yes vs. No* | 1.49 (1.38-1.61) | <0.001 |
| *TCAs, Yes vs. No* | 1.32 (1.22-1.43) | <0.001 |
| *Neurotoxins, Yes vs. No* | 6.41 (5.84-7.04) | <0.001 |
| *No. of preventive classes* | 1.21 (1.15-1.28) | <0.001 |
| Baseline hypertension, Yes vs. No | 0.94 (0.88-1.00) | 0.042 |

Abbreviations: ACEI, angiotensin converting enzyme inhibitor; ARB, angiotensin receptor blocker; TCAs, tricyclic antidepressants.

**Supplemental Figure 1. Covariate Balance between anti-CGRP and Topiramate group**


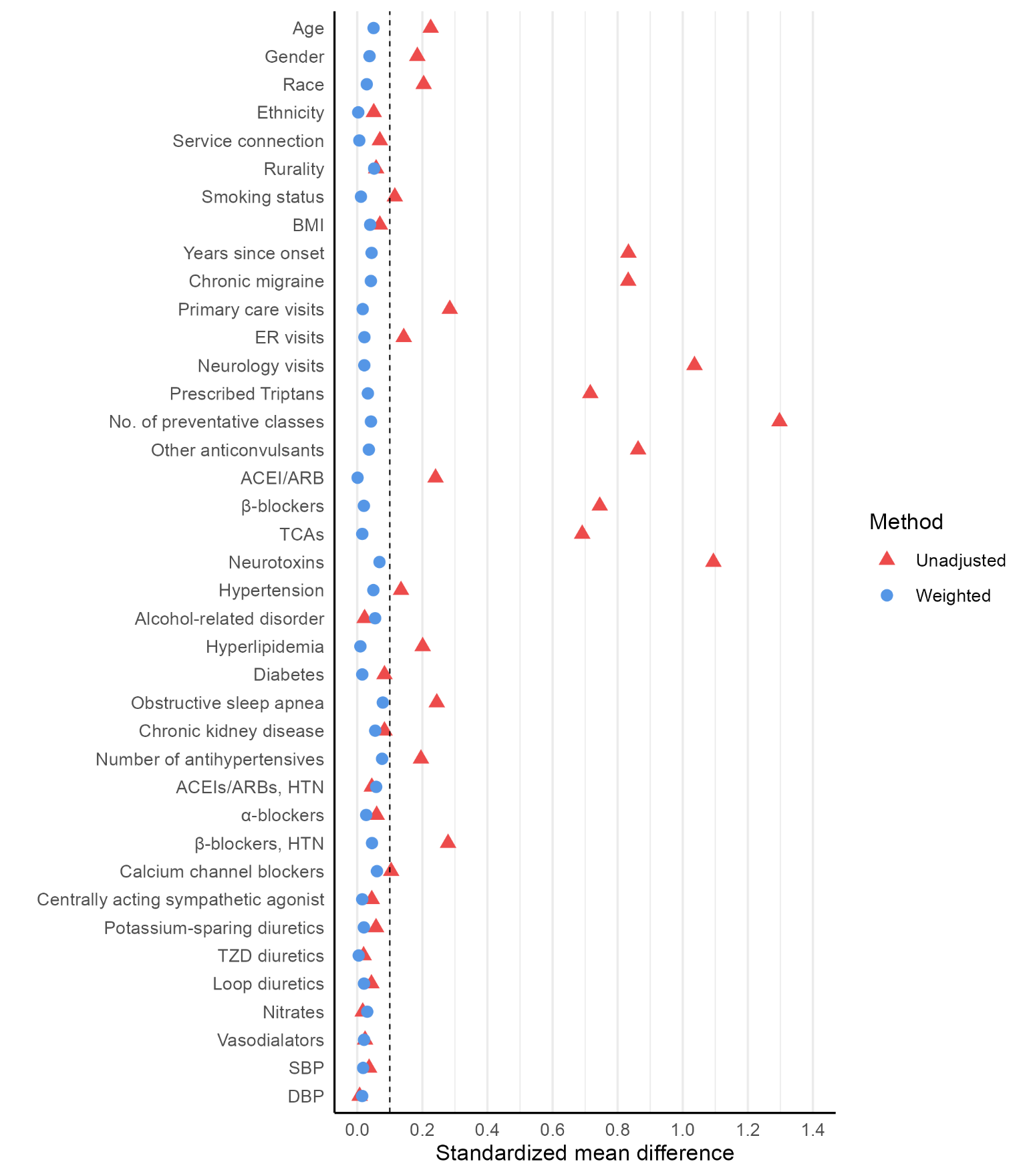


Abbreviations: BMI, body mass index; ER, emergency room; ACEI, angiotensin converting enzyme inhibitor; ARB, angiotensin receptor blocker; TCAs, tricyclic antidepressants; HTN, hypertension; TZD, thiazolidinediones; SBP, systolic blood pressure; DBP, diastolic blood pressure.
